# The changing relationship between Cholera and interannual climate variables in Kolkata over the past century

**DOI:** 10.1186/s13099-023-00565-w

**Published:** 2023-09-13

**Authors:** Debbie Shackleton, Fayyaz Ali Memon, Albert Chen, Shanta Dutta, Suman Kanungo, Alok Deb

**Affiliations:** 1 Centre for Water Systems, Department of Engineering, University of Exeter, EX4 4QF Exeter, UK; 2 National Institute of Cholera and Enteric Diseases, Kolkata, India

**Keywords:** Wavelet Analysis, Cholera, ENSO, IOD, Climate

## Abstract

**Background:**

In the Bengal Delta, research has shown that climate and cholera are linked. One demonstration of this is the relationship between interannual ocean-atmospheric oscillations such as the El Niño Southern Oscillation (ENSO) and the Indian Ocean Dipole (IOD). What remains unclear in the present literature is the nature of this relationship in the specific context of Kolkata, and how this relationship may have changed over time.

**Results:**

In this study, we analyse the changing relationship between ENSO and IOD with cholera in Kolkata over recent (1999–2019) and historical (1897–1941) time intervals. Wavelet coherence analysis revealed significant non-stationary association at 2–4 year and 4–8 year periods between cholera and both interannual timeseries during both time intervals. However, coherence was notably weakened in the recent interval, particularly with regards to ENSO, a result supported by a complementary SARIMA analysis. Similar coherence patterns with temperature indicate it could be an important mediating factor in the relationship between cholera and oscillating climate phenomena in Kolkata.

**Conclusions:**

This study reveals a shifting relationship between cholera and climate variables (ENSO and IOD) in Kolkata, suggesting a decoupling between environmental influences and cholera transmission in recent years. Our results therefore do not suggest that an intensification of ENSO is likely to significantly influence cholera in the region. We also find that the relationship between cholera and interannual climate variables is distinct to Kolkata, highlighting the spatial heterogeneity of the climate-cholera relationship even within the Bengal Delta.

**Supplementary Information:**

The online version contains supplementary material available at 10.1186/s13099-023-00565-w.

## Introduction

Cholera is a highly infectious disease that has plagued humanity for centuries. The Bengal Delta, a vast area consisting of Bangladesh and the Indian state of West Bengal, has been severely affected by endemic cholera, with climate playing a significant role in its persistence. While it is well-established that climate change is likely to exacerbate the occurrence of cholera in the region [1–4], it remains unclear how the relationship between cholera and climate has changed over time. Further, the majority of research has considered the question in relation to Bangladesh. In this article we consider the context of Kolkata, the capital of West Bengal. We seek to answer the question of how the relationship between cholera and interannual climate variables has evolved in the past century in this city, and its implications for the future.

Oscillating interannual climate patterns have previously been implicated in cholera dynamics in Bangladesh [5–7], which constitutes most of the Bengal Delta region. Specifically, in this study we consider the relationships between two interannual climate phenomena: the El Niño Southern Oscillation (ENSO) and the Indian Ocean Dipole (IOD). ENSO is a recurring ocean-atmospheric climate pattern characterized by oscillating sea surface temperature (SST) changes in the Tropical Pacific Ocean that impact weather patterns in the Bengal Delta and across the globe. El Niño events, marked by anomalously warm SSTs, and La Niña events, marked by anomalously cool SSTs, cause changes in the Walker Circulation, an atmospheric system in which westerly “trade winds” across the surface of the Pacific Ocean rise over the western Pacific, causing higher air pressure, and return eastwards aloft [8]. During El Niño events, the Walker Circulation is significantly diminished, resulting in changes to the Bengal Delta’s atmosphere such as higher regional temperatures [9, 10] and reduced precipitation [11, 12]. The IOD refers to a similar oscillating climate phenomenon which occurs within the Indian Ocean and describes the relative changes in SST between the eastern and western sides. The IOD has been shown to be an important modulator of Indian monsoon rainfall and air temperatures across South-East Asia [7, 13].Apart from changes in climate contexts, Kolkata’s water and sanitation context has also evolved over the past century. The Pulta Water Works were established in Kolkata in 1868 to provide a municipal treated potable water supply to the entire city. By 1902, all masonry houses were connected to the mains water supply, and group housing and slums areas were provided with at least one common standpipe. The potable water supply was treated using simple techniques, primarily slow sand filtration and desilting tanks, and was supplied intermittently with an average supply of 109 L per capita per day. In contrast, modern day Kolkata provides a more comprehensive treatment process that includes chlorination and supplies around 240 L per capita per day [14]. Furthermore, the population density has more than doubled from 10,795 population per sq.km in 1911 [15] to 24,252 in 2011 [16]. Moreover, the city’s population growth has led to urban expansion to the East resulting in a greater proportion of the population living further from the Hooghly River. Our hypothesis is that cholera has partially de-coupled from climate over the past century, owing to a reduction in exposure the pathogens in the environment via improved sanitation and water treatment, as well as an increased role of demographic effects, such as over-population.

Wavelet analysis provides a powerful mechanism to quantify the temporal dynamics and non-stationary relationships in epidemiological time series [17]. Its application is particularly effective in analysing the effect of complex climate phenomena like El Niño-Southern Oscillation (ENSO) and Indian Ocean Dipole (IOD), which exhibit interannual variability and whose impacts fluctuate over time. Traditional statistical methods, such as correlation and regression analysis, are ill-suited for these types of data as they assume a stationary relationship over the entire time series, which is rarely the case with such climate indices. In contrast, wavelet analysis can accurately capture and localize the changes in signal frequency and intensity over time, providing an optimal blend of both time and frequency domain information. By incorporating the time-frequency localization property, wavelet analysis permits the detection of changes in periodicity and strength of climate phenomena, and thus, allows for a deeper understanding of the intricate relationships and interactions between different climate variables. This makes it a key tool for evaluating changes in climatic influences over time and enhancing our capability to predict future climate scenarios based on historical data. As a result, wavelet analysis has become a commonly used tool to measure the relationship between climate variables and infectious diseases (e.g. [18–21].).

In this study, we utilize two longitudinal epidemiological datasets for cholera in Kolkata. The first comes from a historical cholera mortality dataset covering the 45-year interval 1897–1941 when the region was part of British India and known as Calcutta. The second is a hospital dataset describing confirmed cholera cases during the 21-year interval 1999–2019. In analysing the time-varying strength of association between cholera and climate variables over these two intervals we will provide evidence that the climate-cholera relationship is non-stationary, suggest mediating factors and discuss its implications for climate change.

## Materials and methods

### Epidemiological dataset

To measure the burden of cholera during the recent interval we used a dataset of stool samples from diarrhoeal patients who reported to the Infectious Disease Hospital (IDH) in Kolkata under their diarrhoeal surveillance system during the 12 years 2008–2019. These data were obtained from the Indian Council of Medical Research - National Institute of Cholera and Enteric Diseases (ICMR-NICED). In the surveillance system, every fifth patient on two randomly selected days of the week (representing around 6% of total patients) were tested for several pathogens including O1 and O139 *Vibrio Cholerae*. We extracted the monthly number of samples which tested positive for either O1 *or* O139 *Vibrio Cholerae*.

For the historical interval, we obtained cholera mortality data from Sanitary Commissioner of Bengal reports from 1897 to 1941 [15]. To account for changes in population, we adjusted these values annually using linear interpolation based on decadal census data from 1891 to 1931. Two years of data were missing from the 1897–1941 dataset (namely 1933 and 1939), which we imputed using seasonally decomposed missing value imputation to create continuous time series datasets necessary for use in wavelet coherence analysis. This method allows the imputed values to retain the seasonal trend present in the rest of the dataset by removing the seasonal component before imputation, then returning the seasonal component.

While the epidemiological dataset used in the historical analysis describes mortality rather than infections, we consider it a reliable proxy for cholera infections over time. While improvements in cholera treatment were made during the historical period, it is unlikely that the majority of the population had access to these treatments, and changes to mortality rates remained consistent at around 60% [22]. Even if a steady decline in mortality rate occurred over the historical period, this likely would have appeared as a gradual trend in the time series signal and would not affect the shorter period frequencies considered in the wavelet analysis.

### Climate dataset

To measure ENSO, we used sea surface temperature (SST) anomaly from the Niño 3.4 region, an equatorial area in the Pacific Ocean bound by the coordinates [5°N-5°S, 170°-120°W], known to characterise well the strength of ENSO [23]. To provide an estimate for this, we extracted monthly SST from Niño 3.4 during the time periods 1897–1941, and 1999–2019 from the Hadley Centre Sea Ice and Sea Surface Temperature (HadISST) dataset (https://psl.noaa.gov/gcos_wgsp/Timeseries/Nino34/) [24]. In order to convert to the standard Oceanic Niño Index (ONI), we first smoothed the data to obtain a 3-month rolling mean. We then de-seasonalised and standardized the data (i.e. taking the temperature anomaly) by subtracting the 50-year monthly mean from the period 1891–1941 for the historical period, and 1970–2020 for the modern period.

To measure the intensity of the IOD, we use the Dipole Mode Index (DMI) which describes the ratio between SST in the western equatorial Indian Ocean [10°S-10°N,50°-70°E] and the south-eastern equatorial Indian Ocean [10°S-0°N,90°-110°E]. This index was extracted directly from NOAA DMI dataset (https://psl.noaa.gov/gcos_wgsp/Timeseries/DMI/) during the same time periods as Niño 3.4 SST.

### Analysis

The wavelet analyses in this study were conducted using the R package {biwavelet} [25]. The wavelet transform, $X(a,\tau )$, describes the contribution of an individual of periodic component with frequency $a$ of a time series signal at time $\tau$ in the form of a ‘mother’ wavelet. The transform is described mathematically as in Eq. 1.

1 $$X\left(a,\tau \right)=\frac{1}{\sqrt{\left|a\right|} }{\int }_{-\infty }^{\infty }x\left(t\right){\psi }^{*}\left(\frac{t-\tau }{a}\right) dt$$

Where $x\left(t\right)$represents the time series under consideration and ^*^ denotes the complex conjugate form. $\psi \left(t\right)$ represents the ‘mother’ wavelet. In our methodology we employed the complex Morlet wavelet [26] which is commonly used in infectious disease modelling due to its ability to produce wavelet transforms with a high frequency resolution [17]. The shape of a Morlet Wavelet is essentially a complex sine wave tapered by a Gaussian and is described by Eq. 2 [27]

2 $$\psi \left(\eta \right)={\pi }^{-\frac{1}{4}}{e}^{i{\omega }_{0}\eta }{e}^{-\frac{{\eta }^{2}}{2}}$$

Here, ${\omega }_{0}$ is the nondimensional frequency of the Morlet wavelet and is taken as 6 (as in [27]) to satisfy the admissibility conditions described in [28].

### Power spectrum

Since the mother wavelet is complex, the resulting transform is also complex. In order to describe the results on the real plane, we therefore consider the real-valued wavelet ‘power’ which is taken as the square of the amplitude.

3 $$Power\left(a,b\right)=\frac{1}{a}\cdot {\left|X\left(a,b\right)\right|}^{2}$$

### Wavelet coherence

In order to quantify the similarity between two time series, we call upon the concept of wavelet coherence [27]. Wavelet coherence analysis uses wavelet transformation to decompose two time-series signals and provides a coherence value between 0 and 1. This value describes the level of correlation between the two decomposed signals where 1 indicates perfect coherence.

Wavelet coherence can be considered analogous to a simple correlation coefficient but measured in the frequency and time domain. This is described by Eq. 4.

4 $$Co{h}_{xy}=\frac{{\left|s\left({X}_{x}\left(a,b\right)\cdot {X}_{y}\left(a,b\right)\right)\right|}^{2}}{s\left(Powe{r}_{X}\left(a,b\right)\right)\cdot s\left(Powe{r}_{y}\left(a,b\right)\right)}$$

### SARIMA analysis

To evaluate the predictive power of ENSO and IOD variables on cholera incidence across various time periods, we employ Seasonal Autoregressive Integrated Moving Average with eXogenous variables (SARIMAX) models. These models improve upon standard regression techniques in time series analysis, primarily due to their ability to manage autocorrelation, as well as potential seasonal or long-term trends, which are common features in time series data. The SARIMAX models accommodate these features through the inclusion of autoregressive components and a moving average. Furthermore, they tackle non-stationarity in time series by implementing a process known as differencing.

Building a SARIMAX model requires the estimation of six parameters: the order of autoregressive terms (p), the degree of differencing (d), the order of the moving average model (q), as well as their seasonal counterparts (P, D, Q). To optimize these parameters, we utilized the auto.arima() function from the {forecast} package in R [29]. This function evaluates multiple parameter combinations and selects the one with the smallest Akaike Information Criterion (AIC), effectively automating the model selection process.

The optimal lag for our models is determined via cross-correlation functions (CCFs) computed between pre-whitened cholera and climate variable time series. The pre-whitening process begins with the automated selection of a suitable SARIMA model for the climate variable. Following this, a univariate SARIMA model - possessing identical parameters to the previously selected one - is applied to the cholera time series for the respective time interval. The residuals of these SARIMA models serve as our pre-whitened time series. The purpose of pre-whitening is to eradicate any misleading correlations that could be influenced by shared trends or autocorrelations in the data.

### Limitations

Our mediation analysis was confined to three variables: temperature, rainfall, and coastal SST.

However, there could be other mediating factors which were not considered in this analysis. For example, sea surface height (SSH) in the Bay of Bengal is known to be strongly influenced to equatorial winds and as such has been linked to both ENSO [30] and IOD [31]. SSH has also been linked to increased cholera cases [32, 33] in the Bengal delta suggesting its potential as a mediating factor. Another plausible mediating factor is chlorophyll concentration in the Bay of Bengal. Positive IOD events have also been demonstrated to increase chlorophyll concentration in the Bay of Bengal due to increased upwelling which leads to more nutrient-rich subsurface waters [34]. This in turn has been shown to increase cholera cases in the Bengal Delta via increased *V.cholerae* concentration [2, 35].

## Results

**Fig. 1:**
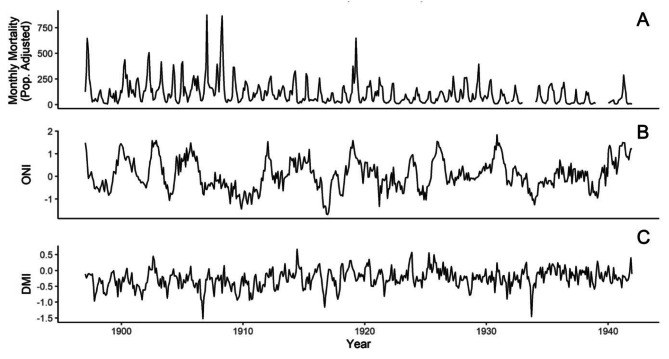
Monthly time series for historical (1897–1941 datasets). **(A)** Cholera deaths in Calcutta adjusted for changes in population. **(B)** Oceanic Nino Index (ONI). **(C)** Dipole Mode Index (DMI)

**Fig. 2:**
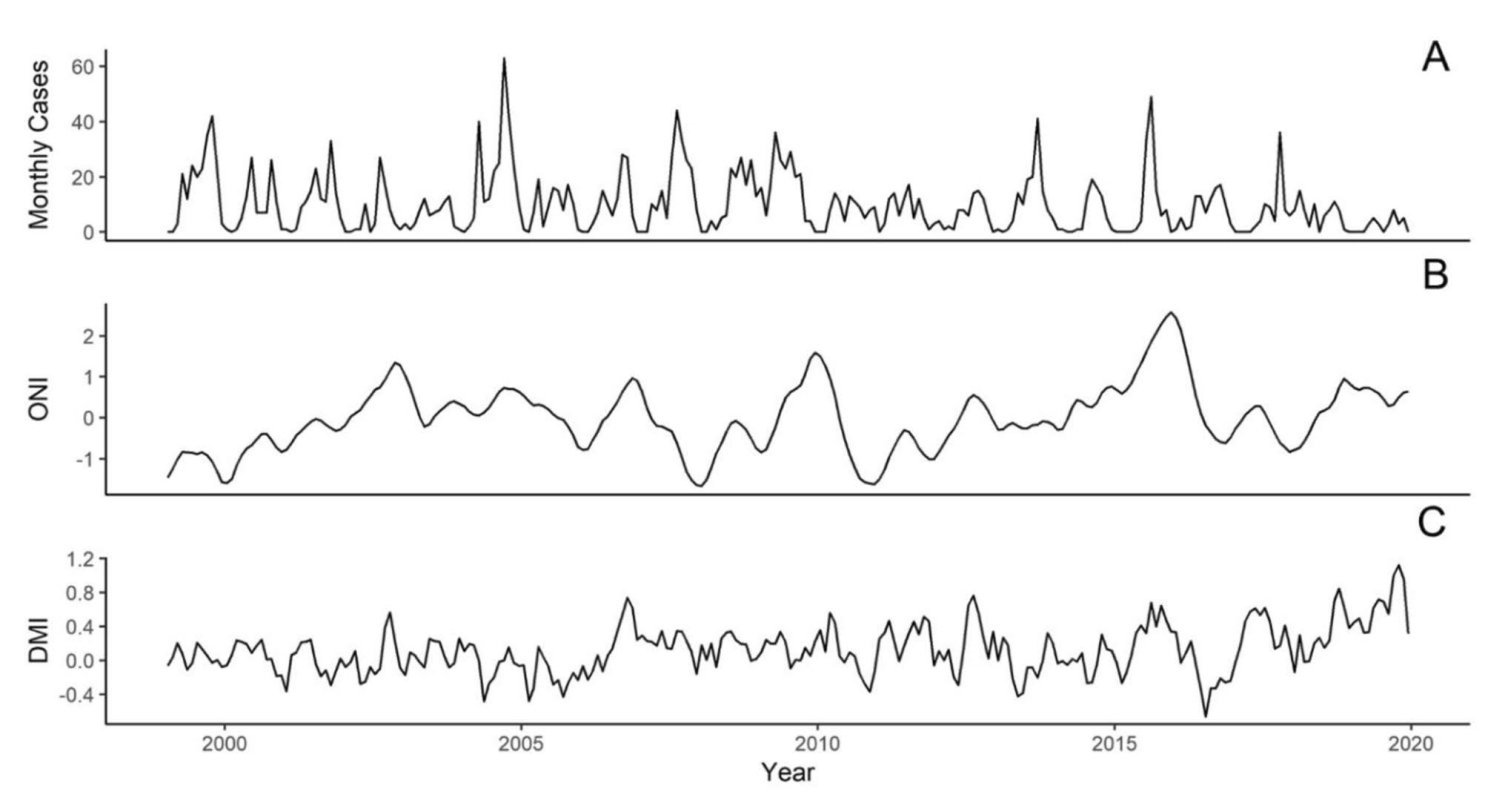
Monthly time series for recent (1999–2019) dataset. **(A)** Recorded cholera cases. **(B)** Oceanic Nino Index. **(C)** Dipole Mode Index

During the historical time period, a total of 79,257 cholera deaths were recorded in Calcutta over 45 years. During the recent time period, a total of 2479 confirmed cholera cases were recorded over 21 years. The time series for cholera, Dipole Model Index (DMI) and the Oceanic Niño Index (ONI) during the two time periods considered are shown in Figs. 1 and 2. The cholera data revealed a persistent seasonal endemic signature with particularly large outbreaks occurring in 1897,1907, 1908, and 1919 during the historical interval, with less pronounced anomalous outbreaks during the recent interval. Additionally, significant shifts in cholera patterns can be observed between the two distinct periods (Figure S2). During the historical interval, cholera mortality was lowest during the monsoon season (July-September), with cases rising post-monsoon and peaking in the summer heat (March-May). However, during the recent interval, the highest incidences of cholera align with the monsoon season, though a secondary, less pronounced peak is also observed in the summer.

**Fig. 3:**
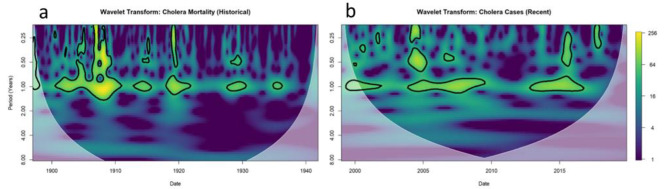
Wavelet power spectrum depicting the strength of a particular wavelet scale across time for historical **(a)** and recent **(b)** epidemiological time signals. Color represents wavelet power level, where yellow regions indicate a strong contribution of a wavelet with a particular period at a particular date. The faded area denotes regions outside of the ‘cone of influence’ where the accuracy of the calculation is reduced by the presence of edge effects. Black outlines denote areas of statistical significance (p < 0.05) against the null hypothesis of white noise

The wavelet transforms identified the dominant mode as annual (Fig. 3) during both time intervals, indicating the seasonal nature of the disease. There are also significant modes at the sub-annual scale for both time intervals. Further, while not statistically significant at the 95% confidence interval, areas of higher periodicity can be witnessed at the 2–4 year period in the recent time interval, and within the 4–8 year period in both time intervals.

**Fig. 4:**
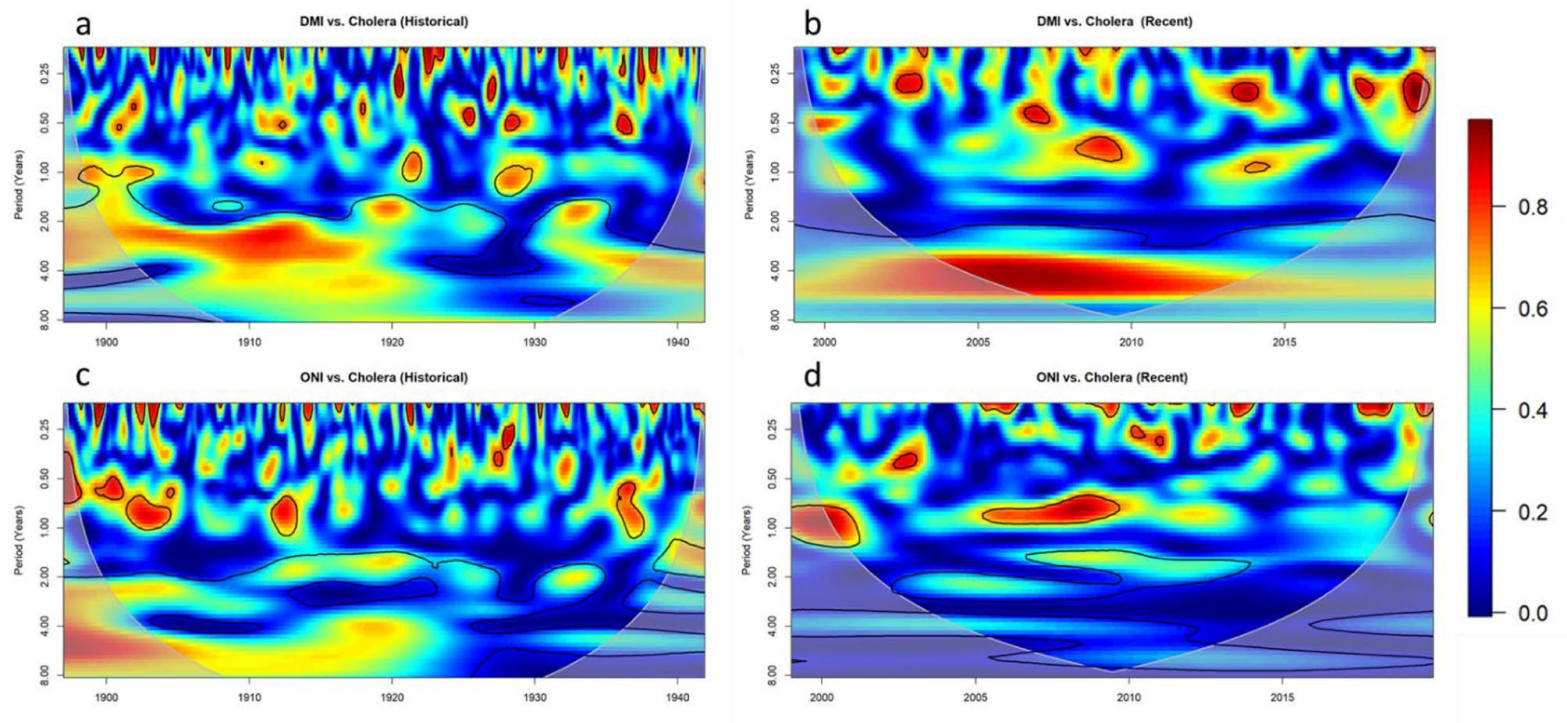
Wavelet coherence bewteen **(a)** DMI and population adjusted cholera mortality during the historical time interval, **(b)** DMI and recorded cholera cases during the recent time interval, **(c)** ONI and population adjusted cholera morality during the historical time interval, and **(d)** ONI and recorded cholera cases during the recent time interval. Colour represents stength of coherence where 1 describes perfect periodicity and 0 describes no relationship. The pale shaded area and black outlines as in (3)

The wavelet coherency between cholera and interannual indices are shown in Fig. 4. The coherence between DMI and cholera is non-stationary but significant during both historical (Fig. 4a) and recent (Fig. 4b) intervals. Significant coherency was observed at 2–8 year periods during all dates considered, with particularly strong coherency (> 0.8) occurring between 1905 and 1915 at the 2–5 year period and between 2005 and 2010 around the 3–6 year periods. With regards to the coherence between ONI and cholera (Fig. 4c), the relationship appears strongest in the earlier part of the historical interval particularly dataset around the 2 year period between 1910 and 1920 and periods between 4 and 8 years from 1915 to 1922. However, after 1922, while periodicity remains significant around the 4 year period and for a short period 1931–1934, the strength of the coherence is much reduced. This reduced coherency remains present in the recent dataset (Fig. 4d) where a weak but significant coherency remains in the 4–8 year period.

**Fig. 5:**
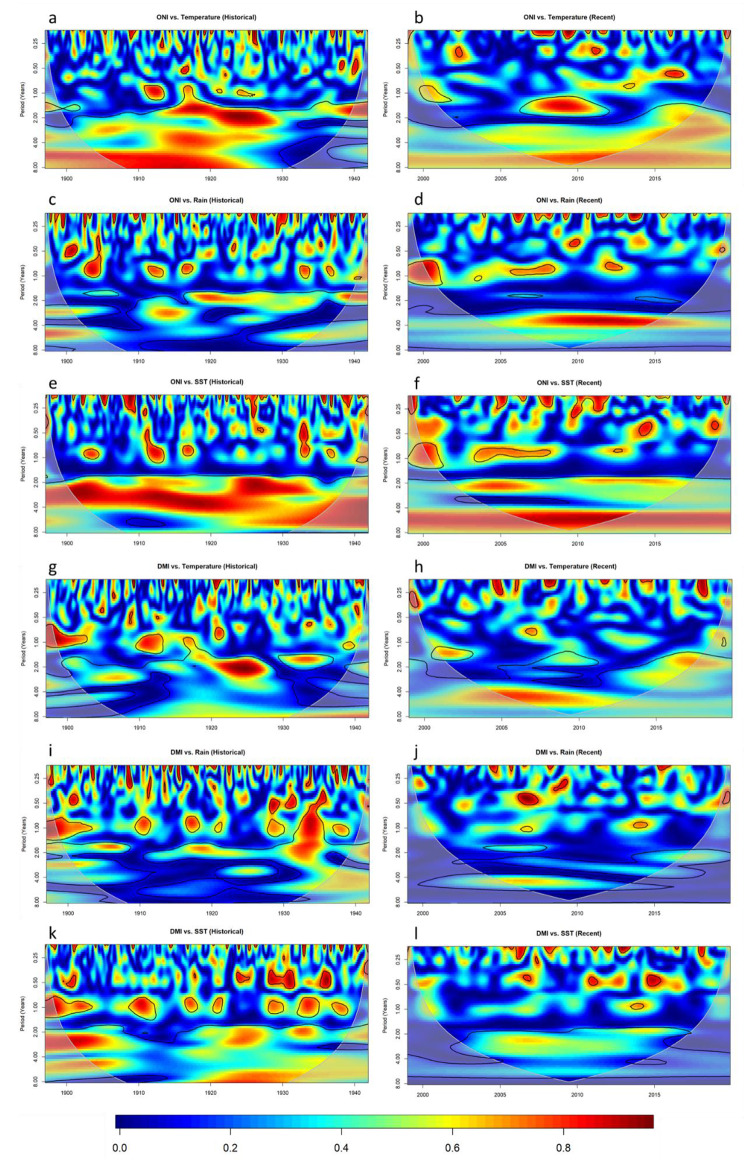
Wavelet coherence between ONI and temperature during the historical interval **(a)** and recent interval **(b)**, ONI and rainfall during the historical interval **(c)** and recent interval **(d)**, ONI and SST during the historical interval **(e)** and recent interval **(f)**, DMI and rainfall during the historical interval **(i)** and recent interval **(j)**, DMI and SST during the historical interval **(k)** and recent interval **(l)**. Colour represents stength of coherence where 1 describes perfect periodicity and 0 describes no relationship. The pale shaded area and black outlines as in (3)

**Fig. 6:**
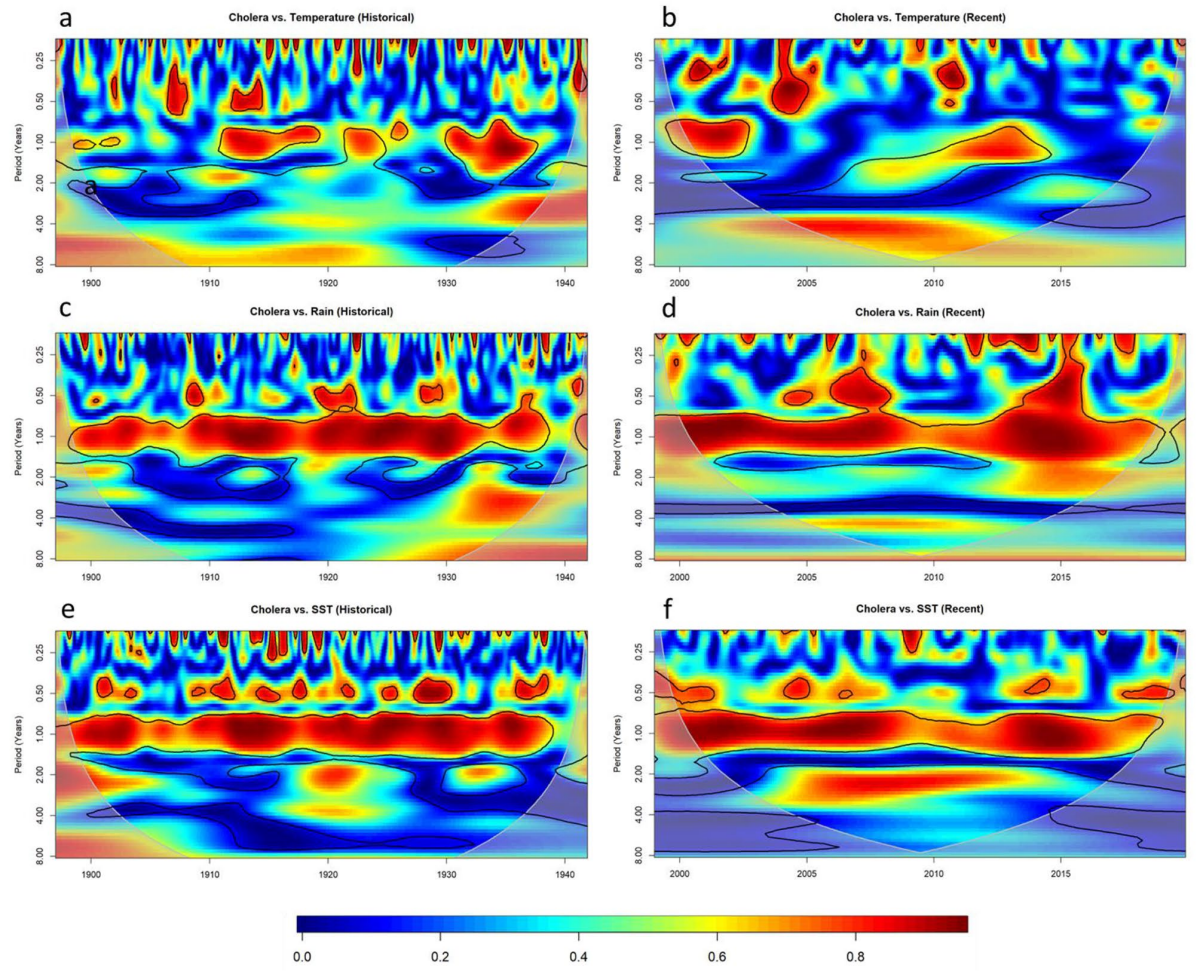
Wavelet coherence between cholera and temperature during the historical interval **(a)** and recent interval **(b)**, cholera and rain during the historical interval **(c)** and recent interval **(d)**, cholera and SST during the historic interval **(e)** and recent interval **(f)**

To delve further into the relationships between cholera and interannual climate variables and consider potential mediating factors, we next considered the relationship between both DMI and ONI and the variables temperature, rainfall, and coastal SST (Fig. 5). The analysis revealed a strong periodicity within the historical dataset between ONI and temperature (Fig. 5a), but not ONI and rainfall (Fig. 5c) or SST (Fig. 5e), within the 4–8 year period where cholera shares periodicity with ONI over the time interval 1905–1925 (Fig. 4C). Further, a highly similar pattern is witnessed in the coherency between temperature and cholera over the historical dataset (Fig. 6a), and is not present in the coherency between cholera and rainfall (Fig. 6c) or SST (Fig. 6e). This suggests that temperature may be a mediating factor in the relationship between ENSO and cholera in the historic dataset. Interestingly, while temperature remains significantly associated with ENSO in the recent time interval, the relationship between cholera and ENSO is much reduced.

Another interesting finding is the strong similarity between the coherency patterns within the recent dataset for DMI and cholera (Fig. 4b), DMI and temperature (Fig. 5h), and temperature and cholera (Fig. 6b). All three plots demonstrate a highly similar oval shape of strong coherency from 2002 to 2012 across the 4–8 year period. This could again suggest that temperature is a mediating factor in the relationship between the IOD and cholera in the recent dataset. Within the historical dataset, despite a significant and reasonably strong (> 0.6) coherency between DMI and cholera during the earlier half of the historical dataset (Fig. 4a), there is limited corresponding coherency between DMI and any of the three weather factors (Fig. 5g,i,k). Our results therefore support the role of temperature as a mediating factor in the relationship between IOD and cholera in the recent period, but do not indicate a mediating factor for the historical period.

The results of our SARIMA analysis largely agree with the results of the wavelet analysis. CCF analysis (Figure S1) found that, during the historical interval, both ONI and DMI showed significant correlations with maximum lags occurring at 2 months (r = 0.10) and 8 months (r = 0.13) respectively. Both variables improved the SARIMA model (Table 1) for historical cholera as regressors, with ONI conferring the greatest improvement with a reduction in AIC of 13.2%. With regards to the recent interval, in line with findings from the wavelet analysis, we found that the ONI/cholera relationship was non-significant at all time lags considered and did not improve the SARIMA model. However, the same insignificance was found between DMI/cholera in contrast to findings from the wavelet analysis.

**Table 1:** Table 1 - Results of SARIMA analysis

Time Interval	Regressor	Model Description	AIC (%change from Null)
Historical	Null	ARIMA(1,0,0)(1,1,0) [11]	962.26
	ONI (lag = 2)	ARIMA(1,0,2)(0,0,1) [11]	834.79 (-13.2%)
	DMI (lag = 8)	ARIMA(1,1,1)(0,0,1) [11]	959.79 (-0.25%)
Recent	Null	ARIMA(1,0,0)(0,1,1) [11]	637.24
	ONI (lag = 12*)	ARIMA(1,0,0)(0,1,1) [11]	639.08 (+ 0.29%)
	DMI (lag = 4*)	ARIMA(1,0,0)(0,1,1) [11]	637.93 (+ 0.1%)

*Indicates not statistically significant at CCF. Null refers to the univariate cholera model. Model description describes the selected SARIMA model parameters in the form ARIMA (p,d,q) (P,D,Q) [m]. Where p refers to the order of the autoregressive terms, d the degree of differencing and q the order of the moving-average model. P,D, and Q describe the equivalent terms for the seasonal part of the model. m refers to the number of time steps in a single seasonal period

## Discussion

Our findings suggest that temperature may be an important factor which mediates the relationship between of cholera and interannual phenomena. This theory is echoed by Pascual et al. [36] who also suggested that the influence of ENSO on cholera in Bangladesh is ultimately mediated by increases in temperature. An association between cholera and temperature has also recently been demonstrated in Kolkata using Generalized Additive Modelling [37]. This is a highly plausible relationship as the causative relationship between temperature and endemic cholera is well-documented in the literature [38]. Laboratory microcosm studies have demonstrated that the bacteria *Vibrio Cholerae* are better able to proliferate in warmer waters [39, 40]. It is argued that this preference brings about an increased concentration of pathogenic *Vibrio Cholerae* bacteria in response to warmer temperatures [3].

An interesting comparison with our results can be found in analysis from Dhaka, an analogous city in Bangladesh, also within the Bengal Delta, 250 km North-East of Kolkata. Research into the association between ENSO and cholera in Dhaka during the historical time interval has found very similar results to our analysis in Kolkata. A study which applied single spectrum analysis (SSA) to ENSO and cholera records in Dhaka from 1893 to 1920 also found a weak association in the earlier half, reducing to uncorrelated in the latter half of the time series [5]. Interestingly, however, the results from both wavelet and SARIMA analysis indicate a much weakened association with ENSO during the recent interval, which differs greatly from previous results in Bangladesh. The same study [5] found that the ENSO/cholera relationship was notably *stronger* and more consistent in a recent time interval (1980–2001) compared with the historical. These results are also in line with a further study in Dhaka [36] which found strong coherence between the time series from cholera and ENSO during the 18-year period from 1980 to 1998. This discrepancy in the ENSO cholera relationship highlights the spatial heterogeneity of the cholera-ENSO relationship within the Bengal Delta [45].

The divergent relationship between the ENSO and cholera in Dhaka and Kolkata during the recent time interval may stem from the differing causes of flooding in these cities. Dhaka, like much of Bangladesh, is situated on a floodplain in the Ganges-Brahmaputra-Meghna (GBM) river basin and as such is frequently exposed to fluvial flooding [41]. It follows that flooding in Dhaka is highly influenced by overall rainfall in the entire GBM catchment region, a vast region encompassing Nepal, Bhutan, Bangladesh and North-East India [42]. Kolkata, while also subject to frequent annual flooding, is generally affected by pluvial flooding caused by a combination of intense urbanisation and heavy localized monsoon rains [43]. Meteorological research has suggested the rainfall-ENSO teleconnection is strongest in more northern (upstream) areas of the GBM basin [44]. This hypothesis would predict that flooding, and consequently flooding induced cholera outbreaks, would hold a stronger association with ENSO in Dhaka compared with Kolkata.

The apparent reduced association between ENSO and cholera in Kolkata in the recent interval compared with the historic interval is largely expected and fits with our hypothesis of de-coupling due to improvements to water and sanitation. We also propose an alternative complementary explanation related to the changes in seasonal patterns. While in both time intervals cholera transmission was present during the summer, cholera incidence from March-May accounted for a much greater proportion of overall burden historically (48%) compared with recently (20%). We believe this could influence the relationships with ENSO in two ways. First, in contracts to monsoon cholera, summer cholera across the Bengal Delta is generally associated with drought [37, 45], which in turn is highly influenced by streamflow and rainfall across the GBM basin [46]. Therefore, the increased teleconnection between rainfall with ENSO in upstream regions of the GBM basin compared with estuarine regions (such as Kolkata) could suggest that historical cholera would hold a greater relationship with ENSO than the more monsoon-centric recent cholera. Second, temperature has been shown to hold a much stronger association with summer cholera than monsoon cholera [36]. Hence, given the mediating role of temperature implied in our study, a decreased role of summer cholera could reduce the overall relationship with temperature, and consequently with ENSO.

Research into the association between cholera and IOD is less well developed, however previous research [47] has found that both positive and negative dipole events are associated with increased cholera incidence in Bangladesh (1983–2008). In a separate study [47], wavelet coherency analysis found a strong association between cholera and IOD in Dhaka during the period 1988–1997, similar to our results in Kolkata during the recent interval. However, the same study found no association in Matlab, a rural area near Dhaka. The findings of a strong IOD/cholera relationship in the dense urban centres of Kolkata and Dhaka, but not in rural Matlab, could suggest that, like ENSO, sensitivity to IOD is strongest is urban cores. An increased climate sensitivity in urban areas is further supported by a study from Reiner et al. [48], and more recently from Perez-Saez et al. [49] who found that the association between cholera and ENSO was much stronger in the central core of Dhaka compared with the more rural peripherals. The authors suggest this could be due to increased vulnerability to flooding as a result of poorer quality housing and greater population density.

The non-stationarity in the relationship between cholera and interannual variables may be explained by the highly complex relationship between ENSO/IOD and weather [12, 50, 51]. For example, a study in Bangladesh found no statistical relationship between ENSO and monsoon rainfall over the period 1948–2012 and found an association with average DMI values only in the Western portion of Bangladesh [52]. This inconsistency is also further demonstrated by the fluctuating associations between interannual climate indicators and the weather variables: rainfall, temperature and SST in Fig. 6. To further complicate matters, when IOD and ENSO events occur simultaneously they can reduce each other’s influence [53]. This non-stationarity of climate-cholera relations well demonstrated by a study form Martinez et al. [54]. A mechanistic model of cholera transmission in Dhaka which incorporated ENSO dynamics predicted a large cholera outbreak in 2016 in response to the 2015–2016 strong El Niño event. However, cholera cases that year were lower than average which the authors speculate was due to improvements to flood controls in the city and a potential decline in the bacterial environmental reservoir caused by a prolonged hiatus of large El Niño events.

With regards to the implications of our finding on the future of cholera in Kolkata, our results suggest a general de-coupling between environment and cholera transmission, particularly with regards to the influence of the ENSO and tentatively with regards to IOD. This suggests that, despite the convenience of early projections of interannual indices, within the context of Kolkata these are likely not helpful for developing cholera early warning systems. Further, our results do not suggest that the projected intensification of ENSO [55] and changes to IOD [56] resulting from climate change will significantly influence cholera burden in Kolkata. However, it is important to note that this analysis does not consider the effects on cholera of climate induced overall changes to air temperature, sea level rise or other individual climate factors which may affect cholera vulnerability.

## Conclusion

This study used wavelet analysis to uncover the dynamic relationship between cholera and interannual climate variables (ENSO and IOD) in Kolkata over historical and recent periods, which reflects the shifting epidemiological patterns of the disease. The results, further validated through SARIMA analysis, reveal a significant shift in cholera patterns and its relationship with these climate variables across the two periods. During the historical period, cholera held a significant and non-stationary relationship with both ENSO and IOD, while in the recent period, the relationship appears to have diminished, especially with regards to ENSO, hinting towards a decoupling between interannual environmental influences and cholera transmission. Our results suggest spatial heterogeneity within the Bengal Delta by demonstrating a different cholera-climate relationship compared with Dhaka which has experienced an increased association with ENSO. We speculate that this may be caused by differing flood mechanisms between the two cities. Further, our analysis suggests that temperature may act as a key mediating factor in these climate-disease relationships. Our results of a de-coupling between cholera and interannual variables indicate that climate-induced changes to ENSO and IOD may not significantly impact cholera burden in Kolkata.

### Electronic supplementary material

Below is the link to the electronic supplementary material.

Supplementary Material 1

## Data Availability

Monthly SST data for the Nino 3.4 region are available from the HadISST dataset at https://psl.noaa.gov/gcos_wgsp/Timeseries/Nino34/. Indian Ocean SST data are available from the NOAA DMI dataset https://psl.noaa.gov/gcos_wgsp/Timeseries/DMI/. The modern epidemiological data is the property of the Indian Council of Medical Research (ICMR) and is not publicly available due to political, privacy and ethical concerns. Historical dataset is publicly available, and can be accessed in digitized from here: https://github.com/DebbieShack/Bengal_cholera_data. Please contact the corresponding author to request the R code files.
