# Supplementary material for: The changing relationship between Cholera and interannual climate variables in Kolkata over the past century
Source: Gut Pathog. 2023 Sep 13;15:42. doi: 10.1186/s13099-023-00565-w (PMC10498578; doi:10.1186/s13099-023-00565-w)
Supplement: Supplementary file 1 — Supplementary Material 1 [file 13099_2023_565_MOESM1_ESM.docx]

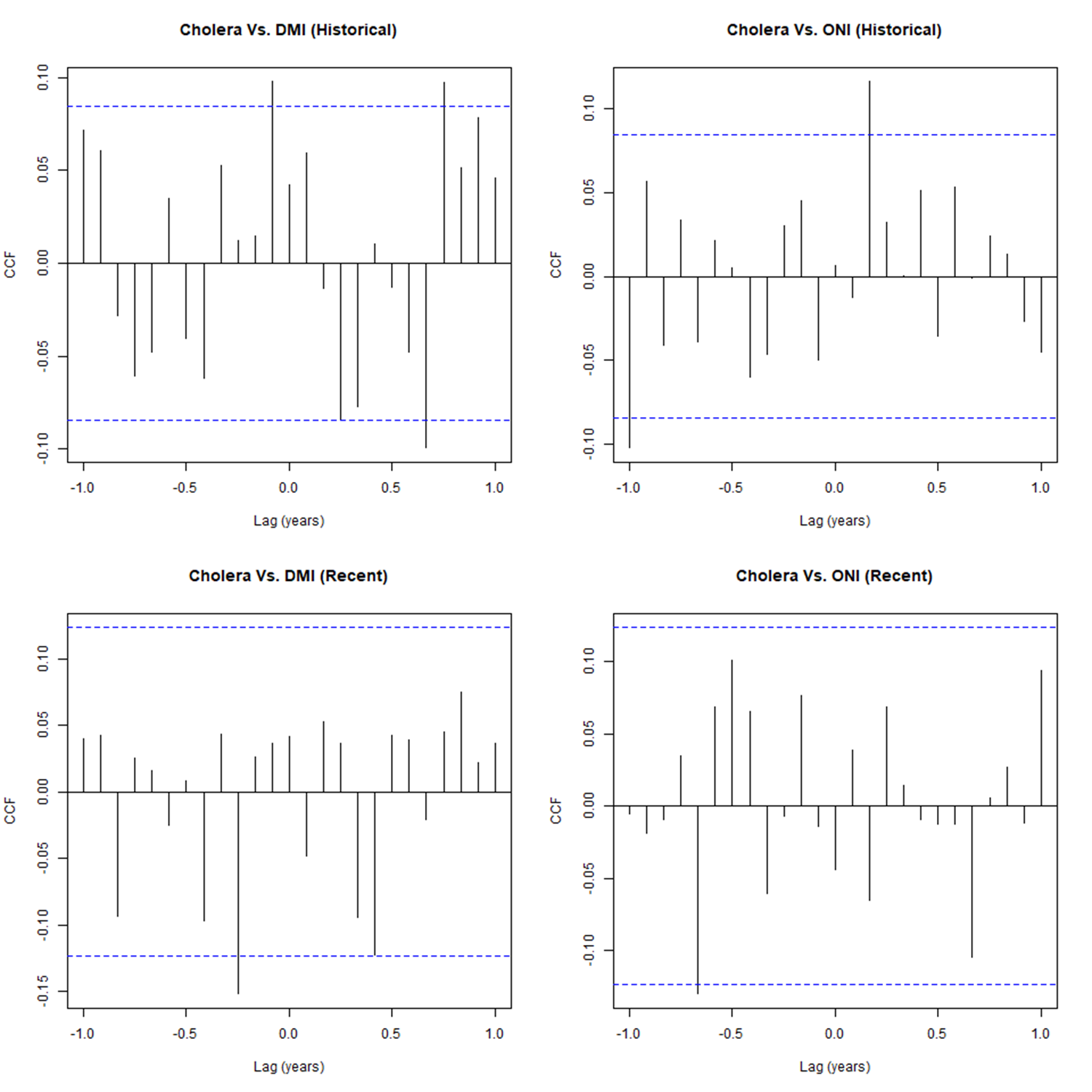


Figure S1. Cross correlation between pre-whitened time series for historical cholera with historical (a) DMI and (b) ONI, and recent cholera with recent (c) DMI and (d) ONI for lag times between -12 and 12 months.


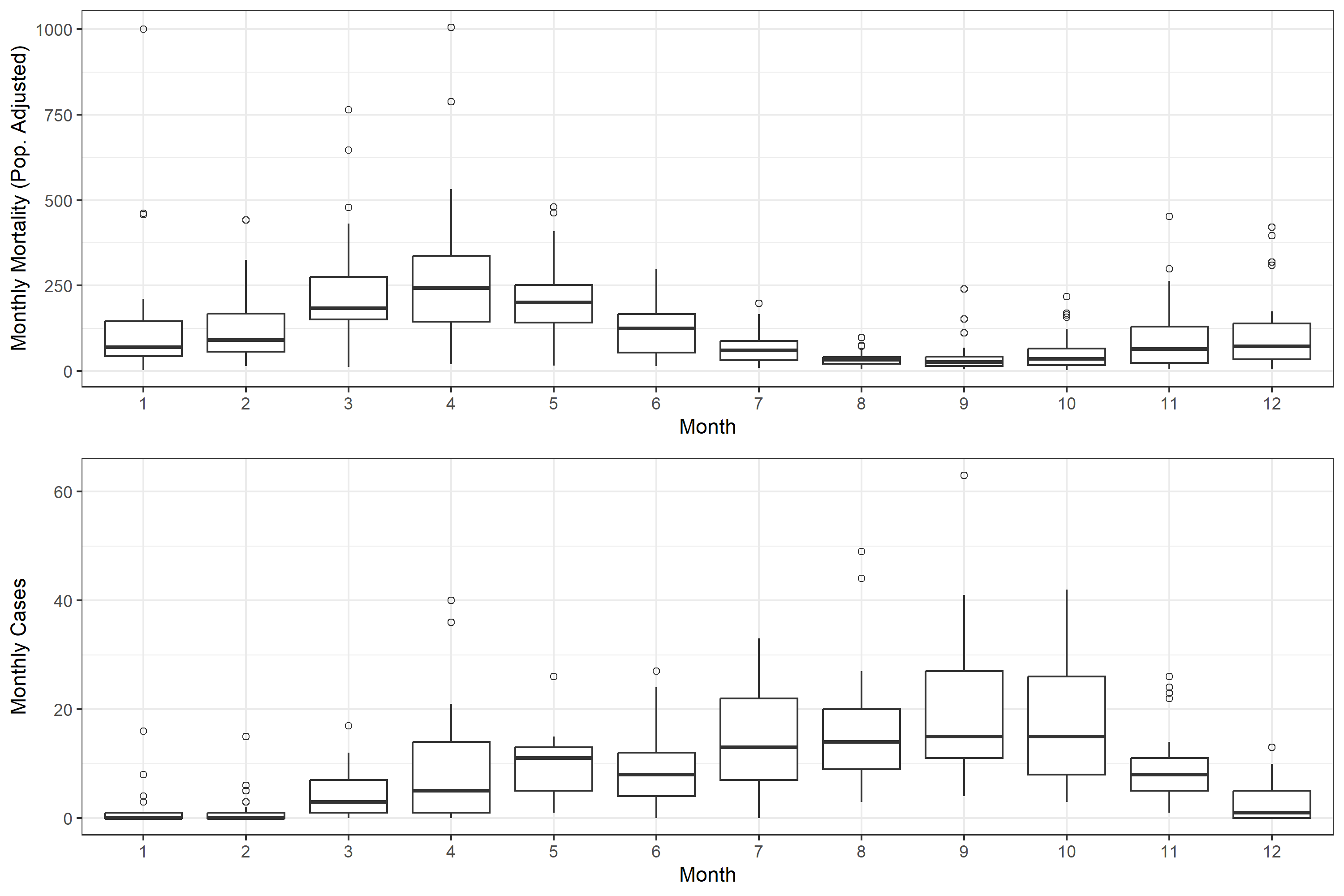


Figure S2. Boxplot showing seasonal patterns in cholera during historical (a) and recent (b) intervals.
